# Effects of a multi-strain probiotic supplement for 12 weeks in circulating endotoxin levels and cardiometabolic profiles of medication naïve T2DM patients: a randomized clinical trial

**DOI:** 10.1186/s12967-017-1354-x

**Published:** 2017-12-11

**Authors:** Shaun Sabico, Ayah Al-Mashharawi, Nasser M. Al-Daghri, Sobhy Yakout, Abdullah M. Alnaami, Majed S. Alokail, Philip G. McTernan

**Affiliations:** 1 Division of Biomedical Sciences, Warwick Medical School, University of Warwick, UHCW Trust, Clifford Bridge Road, Walsgrave, Coventry, CV2 2DX UK; 2 Biochemistry Department, Prince Mutaib Chair for Biomarkers of Osteoporosis, College of Science, King Saud University, PO Box 2455, Riyadh, 11451 Kingdom of Saudi Arabia; 3 School of Science and Technology, Nottingham Trent University, Nottingham, NG1 8NS UK

**Keywords:** Probiotics, Endotoxin, Type 2 diabetes mellitus, Lipids, Clinical trial

## Abstract

**Background:**

The present randomized clinical trial characterized the beneficial effects of a multi-strain probiotics supplementation on improving circulating endotoxin levels (primary endpoint) and other cardiometabolic biomarkers (secondary endpoint) in patients with T2DM.

**Methods:**

A total of 78 adult Saudi T2DM patients (naïve and without co-morbidities) participated in this clinical trial and were randomized to receive twice daily placebo or probiotics [(2.5 × 10^9^ cfu/g) containing the following bacterial strains: *Bifidobacterium bifidum* W23, *Bifidobacterium lactis* W52, *Lactobacillus acidophilus* W37, *Lactobacillus brevis* W63, *Lactobacillus casei* W56, *Lactobacillus salivarius* W24, *Lactococcus lactis* W19 and *Lactococcus lactis* W58 (Ecologic^®^Barrier)] in a double-blind manner for 12 weeks. Anthropometrics and cardiometabolic profiles were obtained at baseline and after 12/13 weeks of treatment.

**Results:**

After 12/13 weeks of intervention and using intention-to-treat analysis, no difference was noted in endotoxin levels between groups [Placebo − 9.5% vs. Probiotics − 52.2%; (CI − 0.05 to 0.36; p = 0.15)]. Compared with the placebo group however, participants in the probiotics groups had a significant but modest improvement in WHR [Placebo 0.0% vs. Probiotics 1.11%; (CI − 0.12 to − 0.01; p = 0.02)] as well as a clinically significant improvement in HOMA-IR [Placebo − 12.2% vs. Probiotics − 60.4%; (CI − 0.34 to − 0.01; p = 0.04)].

**Conclusion:**

Using a multi-strain probiotic supplement daily for 12/13 weeks significantly improved HOMA-IR and modestly reduced abdominal adiposity among medication naïve T2DM patients.

*Trial Registration*: ClinicalTrials.gov Identifier: NCT01765517, Registered January 10, 2013

**Electronic supplementary material:**

The online version of this article (10.1186/s12967-017-1354-x) contains supplementary material, which is available to authorized users.

## Background

In the last few years the gut microbiome has gained considerable interest due to its ability to coexist with its human host and complement several key physiologic processes peacefully maintaining homeostasis and over-all human health [1]. One of the accepted theories that explain the contribution of gut microbes in the development of diseases is sub-chronic inflammation secondary to endotoxemia. This state occurs when fragments of gut-derived Gram negative bacteria (lipopolysaccharides or endotoxin) traverse the intestinal mucosa to enter the circulation, and may represent an important mediator of low-grade systemic inflammation influenced by the host’s own gut microbiota and metabolic state [2]. Previous studies have also shown that endotoxin can stimulate an innate immune response from adipose, liver and skeletal muscle tissues, leading to increased production of pro-inflammatory cytokines [3].

There has been accumulating evidence pointing to the manipulation of the gut microbiome in the prevention and reversal of several chronic non-communicable diseases such as obesity, type 2 diabetes mellitus (T2DM) and the metabolic syndrome [4]. It is now established that dietary intake and nutrition management are significant and effective external factors in modifying the gut ecosystem [5]. Specifically, probiotics, or live bacteria naturally found in the human body that confer health benefits, have shown great potential as adjuvant therapies for a number of insulin-resistant diseases. Currently, randomized clinical trials are limited to strengthen this case. The present investigation aims to fill this gap.

In this study, we determine whether a 12/13 week supplementation of a multi-strain probiotic would induce favorable changes in circulating endotoxin levels (primary outcome) and cardiometabolic profile (secondary outcome) of medication naïve T2DM individuals.

## Methods

The present study is a 12-week single-center, double-blind, randomized, placebo-controlled study. The protocol has been published previously [6] and was registered at the US National Institute of Health (ClinicalTrials.gov Identifier: NCT01765517). Ethical approval was obtained from the Ethics Committee in the College of Science (Approval Code 8/25/16519), King Saud University in Riyadh, Saudi Arabia and written informed consents were obtained from all participants prior to inclusion.

### Subjects

**Fig. 1:**
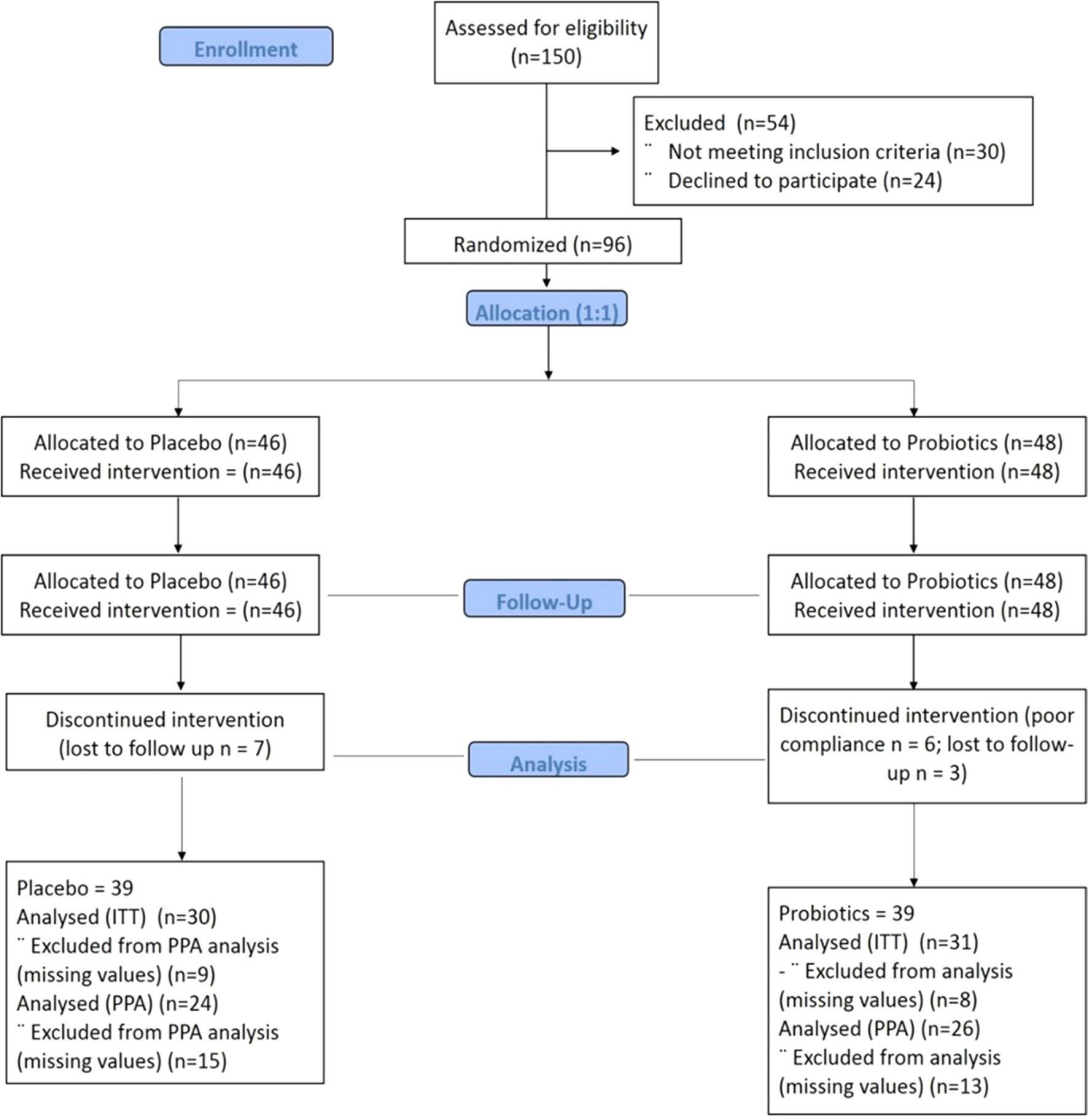
Consort flowchart

All participants were recruited in the outpatient department of King Salman Hospital, Riyadh, Saudi Arabia. A total of 150 adult Saudis (aged 30–60 years old) with newly diagnosed T2DM were initially invited to participate. Participants with T2DM complications and unstable glycemic control were excluded. Those who were already taking probiotics as well as antibiotics, 6 weeks before inclusion, women lactating or pregnant, participants who would anticipate changes in antidiabetic medications (if any) in the next 6 months, on insulin or its analogues and those with gastrointestinal diseases, such as irritable bowel syndrome, were excluded. Thirty participants failed to meet the inclusion criteria and 24 refused to participate. A total of 96 participants were eligible and gave informed consent. They were randomly assigned to either placebo or probiotic sachets unknown to both the principal investigator and the participants. Seventy-eight participants were able to complete the 12 week intervention. A flowchart is shown in Fig. 1.

### Probiotic supplements and allocation

All eligible and consenting participants were given a unique code as identifier. They were allocated (1:1) to receive either probiotics or placebo. The randomization scheme was computer generated by Winclove using permuted blocks with block size equal to 4. It was impossible for research personnel involved with participants to adjust randomization or discern what product participants were receiving, ensuring true allocation concealment. The probiotic group received sachets with 2 g freeze-dried powder of the probiotic mixture Ecologic^®^Barrier (Winclove probiotics, the Netherlands). Ecologic^®^Barrier (2.5 × 10^9^ cfu/g) contains the following bacterial strains: *Bifidobacterium bifidum* W23, *Bifidobacterium lactis* W52, *Lactobacillus acidophilus* W37, *Lactobacillus brevis* W63, *Lactobacillus casei* W56, *Lactobacillus salivarius* W24, *Lactococcus lactis* W19 and *Lactococcus lactis* W58. The placebo group received the same sachets consisting of the carrier of the probiotic product that is maize starch and maltodextrins. The placebo is indistinguishable in color, smell and taste from the probiotic sachets. All participants were asked to consume two sachets per day (dissolving contents in glass of water) once before breakfast and before going to bed.

### Monitoring and blood sample collection

Anthropometry include height (cm), weight (kg), blood pressure (mmHg) waist and hip measurements (cm) were done at baseline and after 12 weeks of intervention. Fasting blood samples were also collected at baseline and after 12 weeks. Samples were immediately centrifuged and serum samples separated. All samples were put in ice and immediately delivered to Prince Mutaib Chair for Biomarkers of Osteoporosis in King Saud University for storage at – 20 °C until further analysis. Participants were asked to return every 4 weeks to surrender unused sachets and to be given fresh refill to monitor compliance. Participants were also asked for any side effects.

### Biochemical analyses

Fasting serum samples were analyzed for glucose and lipid profile (total cholesterol, HDL-cholesterol and triglycerides) using routine analyzer (Konelab, Espoo, Finland). LDL-cholesterol was calculated using the Friedwald equation. Serum insulin and c-peptide were measured using electrochemiluminescence assay (Roche Diagnostics, Germany). Endotoxin was measured using a limulus amebocyte lysate (LAL) quantitative kinetic assay (Lonza, MD, USA). A spike recovery was performed using a sample dilution of 1:40. The recovery spike was 60% and was within the acceptable range of 50–200%. All serum samples were analyzed at baseline and after 12 weeks of intervention.

### Data analysis

Sample size calculation was previously done using endotoxin as primary endpoint [6] and repeated after obtaining actual results. The sample size in the present study (N = 39 per group) has an actual power of 83%. Statistical analysis was performed using Intention-to-treat (ITT) analysis, where missing data were dealt by using the last observation carried forward (LOCF) method. Per-protocol (PPA) analysis was also performed on participants who successfully completed the trial and is presented as a additional data (Additional file 1: Table S1). Data were analyzed using SPSS (version 16.5 Chicago, IL, USA). Mean and standard deviations were used to represent the data for the normal variables, while median and interquartile range were used to report non-normal variables. Furthermore, frequencies and percentages (%) were reported for the categorical data. Changes were also calculated as mean and as percentage. Correlations between endotoxin anthropometrics, glycemic and lipid profile were measured using Spearman correlation coefficient. Independent sample Student T-test and Mann–Whitney U test was used to determine the significant difference between placebo and probiotic groups at baseline. Mixed method analysis of covariance (ANCOVA) was used to determine within and between group differences after adjusting for baseline observations and covariates including WHR, MAP, Glucose (mmol/l), TC/HDL and Endotoxin (IU/ml). All non-normal variables were transformed prior to parametric testing. Intervention effects were presented at 95% confidence interval (CI). p < 0.05 was considered statistically significant.

## Results

Table 1 shows the demographic characteristics of participants assigned to placebo (N = 39) and probiotics (N = 39). No differences were found in age, weight, BMI and sex distribution. The placebo group had significantly higher waist-hip ratio than the probiotics group (p = 0.02). The probiotics group on the other hand, had significantly higher diastolic and mean arterial blood pressure (p values 0.04 and 0.024), respectively. With regards to glycemic and lipid parameters, the probiotics group had significantly higher median glucose levels, as well as circulating levels of total cholesterol and LDL-cholesterol than the placebo group (p < 0.001, 0.001 and 0.05, respectively). The rest of the parameters were not significantly different from one another.

**Table 1:** Baseline parameters according to placebo and probiotics

Parameters	Placebo (N = 39)	Probiotics (N = 39)	p
Males (%)	21 (56.8)	19 (51.4)	
Age (years)	46.6 ± 5.9	48.0 ± 8.3	0.396
Weight (kg)	79.5 ± 15.7	75.6 ± 11.0	0.221
BMI (kg/m^2^)	30.1 ± 5.0	29.4 ± 5.2	0.588
Waist-hip ratio	1.0 ± 0.1	0.9 ± 0.1	0.020
Systolic BP (mmHg)	129.5 ± 10.3	134.8 ± 14.6	0.072
Diastolic BP (mmHg)	78.6 ± 8.6	83.6 ± 11.8	0.040
Mean arterial pressure (MAP)	95.5 ± 7.7	100.7 ± 11.1	0.024
Glucose (mmol/l)	7.0 (5.7 to 11.2)	11.7 (8.4 to 16.4)	< 0.001
Insulin (uU/mL)	13.1 (7.7 to 18.7)	9.9 (7.7 to 16.4)	0.484
C-peptide (ng/ml)	0.1 (0.1 to 0.5)	0.4 (0.0 to 1.8)	0.221
HOMA-IR	4.1 (2.3 to 7.3)	5.3 (3.5 to 10.2)	0.096
Triglycerides (mmol/l)	2.2 ± 1.4	2.5 ± 1.4	0.358
Total cholesterol (mmol/l)	5.2 ± 1.0	5.8 ± 1.3	0.044
HDL-cholesterol (mmol/l)	1.1 ± 0.3	1.0 ± 0.3	0.078
LDL-cholesterol (mmol/l)	3.1 ± 0.9	3.7 ± 1.3	0.051
Total-cholesterol/HDL ratio	5.0 ± 1.3	6.4 ± 2.2	0.001
Endotoxin (IU/ml)	2.1 (1.2 to 4.4)	4.6 (2.4 to 9.9)	0.002

Data presented as Mean ± SD for normal variables while non-normal variables are presented as Median (inter-quartile range)

### Endotoxin

**Fig. 2:**
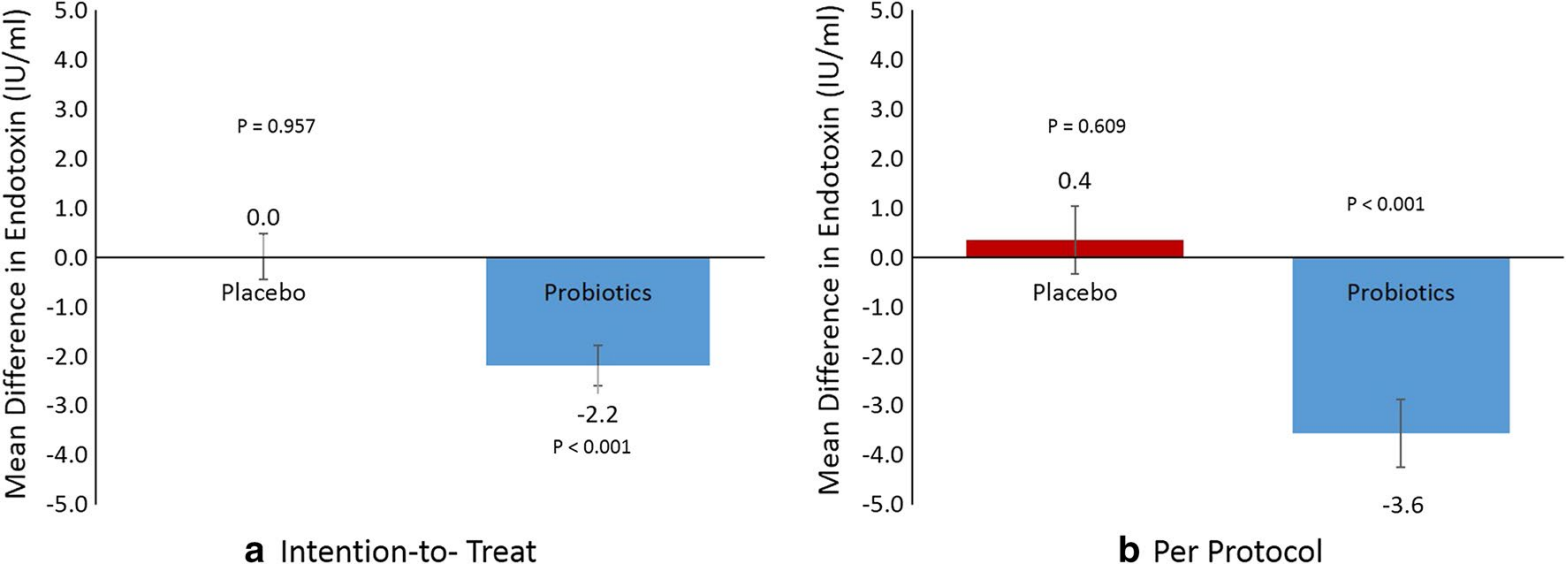
Mean differences in endotoxin levels in placebo versus probiotics using **a** intention-to-treat and **b** per protocol analysis

Within and between group effects of participant characteristics are shown in Table 2. No difference was noted in endotoxin levels between groups [Placebo − 9.5% vs. Probiotics − 52.2%; (CI − 0.05 to 0.36; p = 0.15)]. Within group comparisons however showed a significant decrease in endotoxin levels in the probiotics group (p < 0.01). This was not observed in the placebo group. Mean differences between placebo and probiotics group are presented in Fig. 2.

**Table 2:** Anthropometrics, glycemic and lipid profile characteristics before and after supplementation with placebo or probiotics using intention-to-treat analysis

Parameters	Placebo (N = 39)				Probiotics (N = 39)				Intervention effect	
	Baseline	3-Months	Mean change	P^a^	Baseline	3-Months	Mean change	P^a^	Effect (95% CI)	P^b^
Weight (kg)	79.5 ± 15.7	79.9 ± 15.9	0.42	0.71	75.6 ± 11.0	75.3 ± 11.3	− 0.28	0.77	− 8.00 (− 17.1 to 1.1)	0.08
BMI (kg/m^2^)	30.1 ± 5.0	30.2 ± 5.0	0.15	0.76	29.4 ± 5.2	29.3 ± 5.3	− 0.11	0.86	− 1.27 (− 4.92 to 2.39)	0.49
WHR	1.0 ± 0.1	1.0 ± 0.1	0.00	0.32	0.9 ± 0.1	0.9 ± 0.1	− 0.01	0.75	− 0.07 (− 0.12 to − 0.01)	0.02
SBP (mmHg)	129.0 ± 10.0	130.0 ± 11.0	0.43	0.80	135.0 ± 15.0	129.0 ± 11.0	− 5.84	< 0.01	2.44 (− 5.18 to 10.06)	0.52
DBP (mmHg)	79.0 ± 9.0	80.0 ± 8.0	1.22	0.44	84.0 ± 12.0	80.0 ± 11.0	− 3.78	0.03	0.38 (− 5.82 to 6.58)	0.90
MAP	95.5 ± 7.7	96.5 ± 7.8	0.96	0.60	100.7 ± 11.1	96.2 ± 9.7	− 4.47	< 0.01	1.07 (− 4.58 to 6.72)	0.71
Glycemic profile										
Glu (mmol/l) #	7.0 (5.7 to 11.2)	8.0 (5.9 to 11.4)	1.00	0.02	11.7 (8.4 to 16.4)	8.5 (6.2 to 11.0)	− 3.20	< 0.01	0.05 (− 0.06 to 0.16)	0.36
Ins (uU/mL) #	13.1 (7.7 to 18.7)	10.7 (7.7 to 14.5)	− 2.40	0.72	9.9 (7.7 to 16.4)	6.9 (4.5 to 9.8)	− 3.00	< 0.01	− 0.08 (− 0.24 to 0.07)	0.29
C-Pep (ng/ml) #	0.2 (0.1 to 0.5)	0.2 (0.1 to 0.9)	0.00	0.12	0.5 (0.0 to 1.8)	0.1 (0.0 to 0.3)	− 0.40	0.01	0.08 (− 0.38 to 0.53)	0.74
HOMA-IR #	4.1 (2.3 to 7.3)	3.6 (3.1 to 5.5)	− 0.50	0.37	5.3 (3.5 to 10.2)	2.1 (1.5 to 5.2)	− 3.20	< 0.01	− 0.17 (− 0.34 to − 0.01)	0.04
Lipid profile										
TG (mmol/l)	2.2 ± 1.4	2.0 ± 0.8	− 0.20	0.05	2.5 ± 1.4	1.7 ± 0.7	− 0.78	0.04	− 0.34 (− 0.96 to 0.28)	0.27
TC (mmol/l)	5.2 ± 1.0	4.7 ± 0.9	− 0.53	< 0.01	5.8 ± 1.3	5.1 ± 0.9	− 0.63	< 0.01	0.11 (− 0.52 to 0.75)	0.72
HDL (mmol/l)	1.1 ± 0.3	1.0 ± 0.3	− 0.07	0.46	1.0 ± 0.3	1.1 ± 0.3	0.14	0.10	− 0.04 (− 0.22 to 0.14)	0.65
LDL (mmol/l)	3.1 ± 0.9	2.8 ± 0.9	− 0.37	0.12	3.6 ± 1.3	3.2 ± 0.9	− 0.41	0.02	0.18 (− 0.43 to 0.79)	0.55
TC/HDL	5.0 ± 1.3	4.9 ± 1.4	− 0.11	0.67	6.4 ± 2.2	5.3 ± 4.3	− 1.07	0.35	0.83 (− 0.72 to 2.38)	0.29
Endo (IU/ml) #	2.1 (1.2 to 4.4)	1.9 (1.0 to 2.9)	− 0.20	0.31	4.6 (2.4 to 7.9)	2.2 (1.2 to 3.6)	− 2.40	< 0.01	0.15 (− 0.05 to 0.36)	0.15

Data presented as Mean ± SD for normal variables while non-normal variables are presented as Median (inter-quartile range)

All non-normal variables were transformed prior to parametric testing

*BMI* body mass index, *WHR* waist-hip ratio, *SBP* systolic blood pressure, *DBP* diastolic blood pressure, *MAP* mean arterial pressure, *Glu* glucose, *Ins* insulin, *C-Pep* C-Peptide, *HOMA-IR* homeostasis model for insulin resistance, *TG* triglycerides, *TC* total cholesterol, *HDL* high density lipoprotein, *LDL* low density lipoprotein, *Endo* endotoxin

^#^Median change presented instead of mean

p^a^ and p^b^ denotes p value for within group differences and between group differences respectively obtained from mixed model ANCOVA after adjusting for baseline covariates including WHR, MAP, Glu, (mmol/l), TC/HDL and Endo (IU/ml)

### Anthropometric and clinical measures

Compared with the placebo group, participants in the probiotics groups had a significant improvement in WHR [Placebo 0.0% vs. Probiotics 1.11%; (CI − 0.12 to − 0.01; p = 0.02)]. This significant reduction was also observed using PPA [Placebo 0.0% vs. Probiotics 1.11%; (CI − 0.14 to − 0.03; p = 0.01)] (Additional file 1: Table S1). No differences were noted in weight [Placebo 0.5% vs. Probiotics − 1.4%; (CI − 17.1 to 1.1; p = 0.08)], BMI [Placebo 0.5% vs. Probiotics − 0.4%; (CI − 4.92 to 2.39; p = 0.49)], systolic blood pressure [Placebo 0.3% vs. Probiotics − 0.4%; (CI − 5.18 to 10.06; p = 0.52)], diastolic blood pressure [Placebo 1.5% vs. Probiotics − 4.5%; (CI − 5.82 to 6.58; p = 0.90)] and MAP [Placebo 1.0% vs. Probiotics − 4.4%; (CI − 4.58 to 6.72; p = 0.71)]. Within group comparisons showed a significant decrease in systolic and diastolic as well as mean arterial blood pressure in the probiotics group (p < 0.01, 0.03 and < 0.01, respectively) post intervention. These differences were not observed in the placebo group. Furthermore, no significant changes in weight, BMI and WHR were observed in either group after 3 months (Table 2).

### Glycemic profile

A clinically significant improvement in HOMA-IR was observed in the probiotics group compared to placebo [Placebo − 12.2% vs. Probiotics − 60.4%; (CI − 0.34 to − 0.01; p = 0.04)]. No differences were observed in glucose [Placebo 14.3% vs. Probiotics − 27.4%; (CI − 0.06 to 0.16; p = 0.36)], insulin [Placebo − 18.3% vs. Probiotics − 30.3%; (CI − 0.24 to 0.07; p = 0.29)] and C-peptide [Placebo 0.0% vs. Probiotics 80.0%; (CI − 0.38 to 0.53; p = 0.74)]. Within group comparisons revealed significantly higher glucose levels in the placebo group after 3 months intervention (p = 0.02). In contrast, post-intervention levels of glucose, insulin, C-peptide and insulin resistance were significantly lower than baseline in the probiotics group (p < 0.01, < 0.01, 0.01 and < 0.01, respectively).

### Lipid profile

No differences were observed in all lipid indices: triglycerides [Placebo − 9.1% vs. Probiotics − 31.2%; (CI − 0.96 to 0.28; p = 0.27)], total cholesterol [Placebo − 10.2% vs. Probiotics − 10.9%; (CI − 0.52 to 0.75; p = 0.72)], HDL-cholesterol [Placebo − 6.4% vs. Probiotics − 14.0%; (CI − 0.22 to 0.14; p = 0.65)], LDL-cholesterol [Placebo − 11.9% vs. Probiotics − 11.4%; (CI − 0.43 to 0.79; p = 0.55)] and total/hdl cholesterol ratio [Placebo − 2.2% vs. Probiotics − 16.7%; (CI − 0.72 to 2.38; p = 0.29)]. Within group comparisons showed that both placebo and probiotics group had significantly lower levels of total cholesterol after intervention (p < 0.01). Only the probiotics group, however, showed significantly lower circulating triglycerides and LDL-cholesterol after intervention (p values 0.04 and 0.02, respectively). Both groups had no significant changes in the total/HDL cholesterol ratios post-intervention.

Table 3 shows the bivariate associations between endotoxin and parameters measured. In all participants, endotoxin was significantly associated with diastolic BP (R = 0.27; p = 0.03) and MAP (r = 0.26; p = 0.04). HDL-cholesterol was inversely and significantly associated with endotoxin levels in all participants (R = − 0.25; p = 0.04) and in the probiotics group (R = − 0.35; p = 0.05). In the probiotics group, there were also significant associations between endotoxin and triglycerides (R = 0.37; p = 0.04) and total/HDL cholesterol ratio (R = 0.42; p = 0.02). The latter was also significant in all participants (R = 0.32; p = 0.01).

**Table 3:** Baseline correlations between endotoxin and various parameters

Parameters	ALL (N = 78)		Placebo (N = 39)		Probiotics (N = 39)	
	R	p	R	p	R	p
Age (years)	− 0.06	0.64	− 0.13	0.48	− 0.16	0.40
Weight (kg)	− 0.11	0.38	0.00	0.99	− 0.03	0.86
BMI (kg/m^2^)	− 0.09	0.48	− 0.02	0.91	− 0.06	0.78
Waist-hip ratio	− 0.15	0.27	0.01	0.98	0.03	0.89
Systolic BP (mmHg)	0.25	0.05	0.16	0.40	0.21	0.28
Diastolic BP (mmHg)	*0.27*	*0.03*	0.25	0.19	0.10	0.59
Mean arterial pressure (MAP)	*0.26*	*0.04*	0.22	0.23	0.09	0.63
Glycemic profile						
Glucose (mmol/l)	0.22	0.08	0.15	0.44	− 0.01	0.96
Insulin (uU/ml)	− 0.12	0.35	− 0.18	0.35	− 0.10	0.59
C-peptide (ng/ml)	0.05	0.67	− 0.21	0.27	− 0.04	0.84
HOMA-IR	0.01	0.92	− 0.11	0.56	− 0.11	0.57
Lipid profile						
Triglycerides (mmol/l)	0.21	0.09	− 0.02	0.92	*0.37*	*0.04*
Total cholesterol (mmol/l)	0.19	0.14	0.09	0.64	0.28	0.13
HDL-cholesterol (mmol/l)	*−* *0.25*	*0.04*	0.09	0.63	*−* *0.35*	*0.05*
LDL-cholesterol (mmol/l)	0.14	0.27	− 0.03	0.88	0.23	0.22
Total/HDL cholesterol ratio	*0.32*	*0.01*	0.07	0.73	*0.41*	*0.02*

Data presented as Spearman Correlation coefficients

Italic values indicate significance at p < 0.05

Lastly, none of the participants complained of any serious side effects from the clinical trial. The most common complaint were minor gastrointestinal discomfort (feeling bloated and increased flatulence during the first week of treatment) (N = 5, 1 in the placebo group and 4 in the probiotics group) which is common for first time probiotics users. This symptom gradually faded during the first weeks of treatment.

## Discussion

Individuals with T2DM and those with insulin-resistance in general exhibit higher metabolic endotoxemia than their non-diabetic counterparts [7]. In animal studies it was demonstrated that increased levels of circulating insulin alters intestinal permeability, allowing gut endotoxins to leak in the circulation, which, in turn, initiates a cascade of inflammatory reactions via the innate immune pathway, thus explaining the subclinical inflammation in obesity and insulin-resistant states [8]. Furthermore, a widely accepted theory is that probiotics supplementation can restore a weakened intestinal barrier, preventing endotoxin influx in the circulation and ultimately reducing subclinical inflammation [9]. By manipulating endotoxin levels through the introduction of probiotics in the digestive tract, it is believed that many endotoxin-induced metabolic disorders can be reversed, if not controlled. We conducted our 3-month randomized, double-blind, placebo-controlled clinical trial on the endotoxin-lowering effects of an 8-strain probiotics supplement among participants with T2DM. We found that while circulating endotoxin levels in the probiotics group were no different than placebo after 3 months of intervention, significant improvements in WHR and HOMA-IR were observed.

A recent meta-analysis of RCTs done thus far on probiotics and T2DM revealed that multiple species of probiotics and interventions longer than 8 weeks had stronger metabolic improvements in terms of improved glucose control and lipid profiles [10]. With 8-strains of probiotics supplements used over 3 month duration, we confirm these beneficial effects in reducing abdominal adiposity (measured as WHR) and insulin resistance (HOMA-IR). The lack of improvement in lipid profile however and other indices assessed in the present study do not supersede previous findings. Our results are in agreement with a recent double-blind, randomized trial involving 43 participants (Placebo N = 22 and Probiotic mix N = 21) who were given 8 weeks supplementation of probiotic mix (*Lactobacillus acidophilus* and *casei*; *Lactococcus lactis*; *Bifidobacterium bifidum* and *lactis*; 2 × 10^10^ cfu/day) and found significant reduction in abdominal adiposity with no concomitant decrease in endotoxin levels [11]. Three probiotic species used in the former and the present study, namely, *Bifidobacterium bifidum, Lactobacillus acidophilus and Lactobacillus casei*, have been demonstrated to significantly improve glycemic, inflammatory and lipid profiles of patients with gestational diabetes mellitus after 6 weeks of supplementation [12, 13]. *Lactococcus lactis*, another potent probiotic species used in our study, was recently reported to cure diabetes in non-obese diabetic mice, in combination with low-dose anti-CD3, through a series of actions including decline in insulin autoantibody positivity and stable reversal of hyperglycemia [14]. *Lactobacillus salivarius* was also shown to reverse diabetes-induced intestinal defense impairment through reversal of enteric dysbiosis and decreased endotoxin levels in streptozotocin-induced diabetic mice [15]. Studies using *Lactobacillus salivarius* as a stand-alone probiotic supplement for 4–6 weeks in women with gestational diabetes, however, was not associated with any improvement in metabolic health and pregnancy outcome [16]. In this study, most likely, the cumulative potency of the 8 species employed may have contributed to the significant improvements in the HOMA-IR and WHR of the probiotics group. A recent randomized clinical trial involving 136 Malaysians with T2DM supplemented with either placebo or probiotics (*Bifidobacterium* and *Lactobacillus*) for 12 weeks also showed improvement in terms of glycemic control [17].

We were not able to elicit significant changes in BMI and body weight in both groups studied. This confirms several studies, including the recent meta-analysis by Park and Bae, who concluded limited efficacy of probiotics in weight management [18]. However, clinical trials overall are still very limited and therefore current evidence on probiotics, as weight loss agents are at most, suggestive. We also found no significant improvements in blood pressure although a recent study in animal models showed remarkable improvements in blood pressure after 8 week administration of *Lactobacillus casei* [19]. A recent meta-analysis by Khalesi et al. from 9 clinical trials concluded that probiotic administration may modestly improve blood pressure, and the potency maybe enhanced if multiple species and strains are taken for more than 8 weeks [20].

Despite several trials conducted in the T2DM population, there is still lack in uniformity of findings and this can be due to discrepancies in sample size, duration of treatment, different inclusion criteria and type of analyses done. A major potential source of conflicting results from different studies however maybe from the omission of strain-specific information from past observations. Studies have shown that effects of probiotics are often species-, or strain/strains-specific [21, 22]. Hence, information on strain-specific mechanisms for the probiotics used are essential. In the present study, the rationale for choosing the multiple-strain probiotics for the clinical trial were based on a series of in vitro experiments performed on the probiotic strains’ ability to strengthen the epithelial barrier, which, as previously hypothesized, is necessary to reduce circulating endotoxin, the primary endpoint. More specifically, the mechanisms found among these strains include (a) in vitro strengthening of the epithelial barrier after a pathogenic bacteria stressor (*B. lactis* W52, *L. casei* W56 and *Lc. Lactis* W58) and/or after an inflammatory stressor (*B. bifidum* W23, *L. acidophilus* W37, *L. brevis* W63, *L. casei* W56 and *Lc. Lactis* W19), (b) inhibition of mast cell activation (*B. bifidum* W23, *B. lactis* W52, *L. casei* W56 and *L. salivarius* W24), (c) stimulation of anti-inflammatory cytokines (all strains except *L. salivarius* and *Lc. Lactis* W19) and (d) decreasing lipopolysaccharide load (*B. bifidum* W23, *L. acidophilus* W37 and *Lc. Lactis* W19) [23].

The authors acknowledge several limitations of this study. Gut microbiome analysis was not measured, therefore, successful colonization of these strains in the intestinal tract cannot be confirmed. Dietary intake and physical activity of all participants were also not monitored and this could explain beneficial changes in the placebo group. Despite randomization and blinding, there were still significant differences between placebo and probiotics group at baseline. While this was addressed by adjusting analyses for baseline differences, the additional adjustments of covariates made it more difficult to elicit the desired treatment effect because of the added statistical stringency to the cohort. This is worth highlighting because the probiotics group made a more substantial improvement and hence the disparity with the placebo. The study’s strengths include it’s randomized, double-blind, placebo-controlled design and well defined cohort. To the best of our knowledge this is also the first trial on probiotic supplementation in the Arabic T2DM population. This is important since the gut microbiome, although mostly populated by *Firmicutes* and *Bacteroidetes* is highly affected not only by the health status of the individual, but more so by geography and ethnicity [24]. Despite the large dropout rate from participants, the study remained sufficiently powered and adequately blinded.

## Conclusions

In summary, a 12-week, multi-strain probiotic supplementation in medication naïve T2DM individuals resulted in no significant changes in circulating endotoxin levels but has been beneficial in terms of improved HOMA-IR and modest reduction in abdominal adiposity. A larger cohort and a longer duration of treatment may be necessary to investigate if probiotic supplementation can be protective against diabetic complications.

## Additional file

**Additional file 1: Table S1.** Anthropometrics, glycemic and lipid profile characteristics before and after supplementation with placebo or probiotics using per protocol analysis.

## Abbreviations

ANCOVA: analysis of covariance
BMI: body mass index
HOMA-IR: homeostasis model assessment of insulin resistance
ITT: intention-to-treat
LAL: limulus amebocyte assay
LCOF: last observation carried forward
MAP: mean arterial pressure
PPA: per protocol analysis
RCT: randomized clinical trial
T2DM: type 2 diabetes mellitus
WHR: waist-hip ratio
