# Supplementary material for: Effects of a multi-strain probiotic supplement for 12 weeks in circulating endotoxin levels and cardiometabolic profiles of medication naïve T2DM patients: a randomized clinical trial
Source: J Transl Med. 2017 Dec 11;15:249. doi: 10.1186/s12967-017-1354-x (PMC5725828; doi:10.1186/s12967-017-1354-x)
Supplement: Supplementary file 1 — Additional file 1: Table S1. Anthropometrics, glycemic and lipid profile characteristics before and after supplementation with placebo or probiotics using per protocol analysis. [file 12967_2017_1354_MOESM1_ESM.doc]

**Supplementary Table 1.** Anthropometrics, Glycemic and Lipid Profile Characteristics Before and After Supplementation with Placebo or Probiotics Using Per Protocol Analysis

| **Parameters** | **Placebo (N=24)** | | | | **Probiotics (N=26)** | | | | **Intervention Effect** | |
| --- | --- | --- | --- | --- | --- | --- | --- | --- | --- | --- |
|  | Baseline | 3-Months | Mean Change | P^a^ | Baseline | 3-Months | Mean Change | P^a^ | Effect (95% CI) | P^b^ |
| Weight (kg) | 79.5 ± 15.7 | 79.9 ± 15.9 | 0.42 | 0.43 | 75.5 ± 10.9 | 75.4 ± 11.4 | -0.07 | 0.66 | -2.05 (-11.92 – 0) | 0.72 |
| BMI (kg/m^2^) | 30.1 ± 5.0 | 30.2 ± 5.0 | 0.15 | 0.96 | 29.4 ± 5.2 | 29.3 ± 5.4 | -0.03 | 0.98 | -0.77 (-4.67 - 3.14) | 0.69 |
| WHR | 0.9 ± 0.1 | 0.9 ± 0.1 | 0.00 | 0.18 | 0.9 ± 0.1 | 0.9 ± 0.1 | -0.01 | 0.65 | -0.08 (-0.14 - -0.03) | 0.01 |
| SBP (mmHg) | 129.5 ± 10.8 | 130.1 ± 11.7 | 0.57 | 0.65 | 132.7 ± 13.7 | 129.8 ± 12.7 | -2.82 | 0.25 | -2.44 (-11.93 - 7.05) | 0.61 |
| DBP (mmHg) | 78.4 ± 9.1 | 80.0 ± 8.5 | 1.63 | 0.38 | 83.2 ± 12.4 | 80.0 ± 11.7 | -3.24 | 0.15 | 2.51 (-5.97 - 10.98) | 0.55 |
| MAP | 95.4 ± 8.5 | 96.7 ± 8.6 | 1.28 | 0.59 | 99.7 ± 11.2 | 96.6 ± 10.7 | -3.10 | 0.11 | 0.86 (-6.77 - 8.49) | 0.82 |
| Glycemic Profile | | | | | | | | | | |
| Glu (mmol/l) # | 7.1 (5.7 - 11.2) | 8.0 (5.9 - 11.4) | 0.90 | 0.01 | 11.7 (8.4 - 16.4) | 8.5 (6.2 - 10.9) | -3.20 | <0.01 | 0.06 (-0.06 - 0.19) | 0.30 |
| Ins (uU/mL) # | 13 (7.5 - 18.7) | 10.9 (7.7 - 15.5) | -2.10 | 0.49 | 9.8 (7.7 - 15.2) | 6.8 (4.5 - 9.6) | -3.00 | 0.04 | -0.08 (- 0.28 - 0.12) | 0.41 |
| C-Pep (ng/ml) # | 0.2 (0.1 - 0.4) | 0.3 (0.1 - 0.9) | 0.10 | 0.16 | 0.7 (0.0 - 2.0) | 0.1 (0.0 - 0.3) | -0.60 | 0.01 | 0.07 (-0.43 - 0.56) | 0.79 |
| HOMA-IR # | 4.1 (2.3 - 7.5) | 3.6 (3.1 - 6.0) | -0.50 | 0.29 | 5.2 (3.5 - 10.2) | 2.1 (1.5 - 4.4) | -3.10 | <0.01 | -0.18 (-0.39 - 0.04) | 0.11 |
| Lipid Profile | | | | | | | | | | |
| TG (mmol/l) | 2.1 ± 1.4 | 2.0 ± 0.8 | -0.20 | 0.05 | 2.5 ± 1.4 | 1.7 ± 0.7 | -0.78 | 0.15 | -0.41 (-1.13 - 0.31) | 0.26 |
| TC (mmol/l) | 5.2 ± 1.0 | 4.7 ± 0.9 | -0.53 | 0.00 | 5.8 ± 1.3 | 5.1 ± 0.9 | -0.63 | <0.01 | 0.30 (-0.39 - 0.98) | 0.39 |
| HDL (mmol/l) | 1.1 ± 0.3 | 1.0 ± 0.3 | -0.07 | 0.15 | 1.0 ± 0.3 | 1.1 ± 0.3 | 0.15 | 0.20 | -0.10 (-0.30 - 0.10) | 0.31 |
| LDL (mmol/l) | 3.2 ± 0.9 | 2.8 ± 0.9 | -0.41 | 0.11 | 3.7 ± 1.2 | 3.3 ± 0.9 | -0.44 | 0.03 | 0.43 (-0.24 - 1.10) | 0.21 |
| TC/HDL | 5.0 ± 1.3 | 4.9 ± 1.4 | -0.13 | 0.88 | 6.5 ± 2.2 | 5.4 ± 4.4 | -1.08 | 0.41 | 1.33 (-0.39 - 3.05) | 0.13 |
| Endo (IU/ml) # | 2.3 (1.2 - 4.6) | 2.0 (1.1 – 4.7) | -0.30 | 0.14 | 5.1 (3.2 - 8.4) | 2.3 (1.2 - 3.6) | -2.80 | <0.01 | 0.20 (-0.05 - 0.45) | 0.12 |

**Note**: Data presented as Mean ± SD for normal variables while non-normal variables are presented as Median (inter-quartile range); #median change presented instead of mean; all non-normal variables were transformed prior to parametric testing; p^a^ and p^b^ denotes p-values, for within group differences and between group differences respectively, obtained from mixed model ANCOVA after adjusting for baseline covariates including WHR, MAP, Glu (mmol/l), TC/HDL and Endo (IU/ml). BMI, body mass index; WHR, waist-hip ratio; SBP, systolic blood pressure; DBP, diastolic blood pressure; MAP, mean arterial pressure; Glu, glucose; Ins, insulin; C-Pep, C-Peptide; HOMA-IR, homeostasis model for insulin resistance; TG, triglycerides; TC, total cholesterol; HDL, high density lipoprotein; LDL, low density lipoprotein; Endo, endotoxin.
